# A Review of the Components of Problematic Exercise in Psychometric Assessment Instruments

**DOI:** 10.3389/fpubh.2022.839902

**Published:** 2022-03-31

**Authors:** Álvaro Sicilia, Manuel Alcaraz-Ibáñez, Adrian Paterna, Mark D. Griffiths

**Affiliations:** ^1^Health Research Centre, Department of Education, University of Almería, Almería, Spain; ^2^Psychology Department, Nottingham Trent University, Nottingham, United Kingdom

**Keywords:** exercise addiction, exercise dependence, compulsive exercise, excessive exercise, obligatory exercise, commitment to exercise

## Abstract

**Backgrounds:**

The range of theoretical conceptualizations of problematic exercise in psychometric assessment instruments makes it difficult to identify the components that define this phenomenon. A better understanding of the underlying components of problematic exercise may contribute to progress toward providing scientific evidence that allows for deciding whether problematic exercise should be considered a substantive mental health disorder. The objective of the present review was to examine and compare, through a content analysis of their items, the components of problematic exercise in psychometric assessment instruments identified in a recent systematic review.

**Methods:**

A total of 33 components of problematic exercise were identified in the 17 assessment instruments included in the present review.

**Results:**

The results show that, despite the lack of consensus in the operational definition of their factors and the variety of ways of wording their items, the instruments reflect some common components that might indicate core criteria (i.e., salience, withdrawal, and mood modification) or candidate components (i.e., conflict, and continuance despite problems) of problematic exercise. However, other components of different nature were shown to be specific to some of the problematic exercise conceptualizations on which the assessment instruments are based.

**Conclusion:**

In the interest of reaching a consensus that allows to advance in this research field, further studies are needed to resolve which components are inherently problematic.

## Introduction

Problematic exercise broadly refers to exercising in a way that the individual loses control over the behavior, so that it begins to have negative physical, psychological, and social consequences (1). Despite the possible negative effects that problematic exercise can have (2), this behavior has not been recognized to date as a mental health disorder in leading clinical manuals (3, 4). One of the main causes behind this lack of recognition is the insufficient scientific evidence to establish the diagnostic criteria and course descriptions needed to identify this behavior as mental health disorder (3).

Most survey research examining problematic exercise has been conducted using psychometric assessment instruments (5). However, the fact that the instruments for problematic exercise utilize different terminology and theoretical conceptualizations (6) makes it difficult to identify the essential components that should define this phenomenon. Without a clear consensus on the components that should define problematic exercise, it is difficult to compare the results of the studies and, therefore, to show scientific evidence that helps to establish the diagnostic criteria and course description needed to identify problematic exercise as a disorder (3). Determining core components that define problematic exercise is a central task for its description as a disorder, but also for its subsequent prevention and treatment. In addition, psychometric assessment instruments form the basis of evidence reported in prevalence studies of problematic exercise, so when these instruments vary in their definitions and operational components it becomes difficult to understand the nature of this phenomenon (7). Examination of the components of problematic exercise in the assessment instruments would allow comparisons to be made between them and a future consensus to be established on the definition of problematic exercise.

Colledge et al. (7) conducted a brief review of the assessment instruments for problematic exercise currently in use, showing the existence of a variety of instruments with different theoretical conceptualizations. The three most widely used instruments were the Exercise Dependence Scale [EDS, (8)], which defines the problematic exercise based on criteria for substance dependence provided by the DSM-IV (9), the Exercise Addiction Inventory [EAI, (10)], that operationalizes the problematic exercise based on the components model of behavioral addictions (11), and the Compulsive Exercise Test [CET, (12)], where problematic exercise is defined as a means of regulating body size and weight based on a cognitive behavioral conceptualization (13).

Recently, Sicilia et al. (6) conducted a systematic review to examine the theoretical conceptualizations of problematic exercise in psychometric assessment instruments. The findings from this study also showed a variety of theoretical conceptualizations of problematic exercise and demonstrated a lack of consensus concerning its definition. The authors classified the instruments according to their conceptualization into five groups: (i) problematic exercise as an end of an exercise continuum, (ii) problematic exercise as a behavioral addiction, (iii) problematic exercise as a dependence, (iv) problematic exercise as a means of regulating body size and weight, and (v) no clear conceptualization. However, the authors highlighted a strong dichotomy in relation to the primary nature (i.e., a problematic exercise irrespective of whether other disorders may occur) or secondary nature (i.e., the concern with exercise is not better accounted for by other disorders) of problematic exercise, which could limit the ability of the instruments to adequately capture the dimensionality of this construct. Therefore, although it has been suggested that problematic exercise may have different etiologies (14, 15), research has also shown overlaps between these ways of defining problematic exercise (16, 17). Consequently, Sicilia et al. (6) recommended that, in addition to qualitative studies, future research should undertake comparative analyses of the components or criteria covered in the psychometric assessment instruments of problematic exercise, such as has been carried out on other potentially problematic behaviors, such as gaming and pornography use (18, 19).

An examination of the items included in the instruments assessing problematic exercise would provide greater insight on the nature of the components proposed for such deleterious behavior. Furthermore, the identification of common and specific components in instruments with different theoretical conceptualizations would help to interpret the results derived from different instruments. Therefore, taking up the recommendation made in the systematic review by Sicilia et al. (6), the present study significantly extends that review and, using content analysis, aims to identify, examine, and compare the components of problematic exercise proposed in the psychometric assessment instruments identified in that review. The present study assumes the generic term “problematic exercise” in the form used by Sicilia et al. (6), in such a way that the authors do not intend to position themselves *a priori* on any of the perspectives or theoretical models on which the instruments are based, but rather to examine and compare, in an exploratory manner, the components assessed by those instruments.

## Methods

In the present study, we examined the items included in the 17 instruments assessing problematic exercise identified in a recent systematic review conducted by the present authors [for (6)]. The first and third authors coded the data on the characteristics of the studies identified by Sicilia et al. (6) using a coding sheet (see Appendix A). Disagreements in the data coding procedure were resolved by discussion between the two authors. Data from the studies were classified into the following categories: (i) instrument; (ii) author(s); (iii) sample characteristics; (iv) conceptualization; (v) instrument structure; and (vi) factors and definition (see Appendix B).

Second, based on similar methodology to that used by King et al. (18) and Fernández and Griffiths (2), the psychometric instruments included in the study selection were compared on their ability to assess different components utilizing a coding procedure of their items (20). This analysis entailed moving from the text included in the items to their common thematic elements. This procedure was developed through different phases. In the first phase, the items of the assessment instruments for problematic exercise were collected and a previous immersion with repeated reading of the items was performed. Subsequently, the research team proceeded to search for, identify, and label the components according to the thematic content represented in each of the items (20). This was achieved by combining two methods: deductively considering criteria from the already established theory or manual, and inductively observing the components that emerged in the items in those cases that their wording expressed a concept that did not match with any established criteria in literature. In the latter case, the theme that emerged from the analysis of the item's content was observed and a new component or element was proposed. Enough items were coded by first and second authors until the emerging components of problematic exercise instruments were agreed and defined (see Appendix C). Following this, the first and second authors coded all items of the instruments according to the components established previously by agreement using an Excel spreadsheet. Likewise, some items were coded on more than one addiction component when it appeared to be assessing more than one component. Disagreements in the content analysis of items were resolved by discussion between the first two authors. In addition, all items were independently coded by the fourth author. Discrepancies were reconciled by revisiting the wording of items and reaching a consensus among authors. Finally, the results were ordered in the form of a table (see Table 2), designed to show the problematic exercise components that emerged in each of the assessment instruments considered in the present review.

## Results

The assessed components, definitions and example of item are shown in Appendix C. The comparison of instruments utilizing the same definition to each component provides a consistent base on which to examine similarities and differences between the instruments in terms of their assessed components. A comparison of the components assessed in the three instruments most frequently used in the recent literature (7, 21) is shown in Table 1.

**Table 1:** Comparison of components assessed by the EDS, EAI and CET.

	**EDS**		**EAI**		**CET**	
**Assessed component/s**	**Instrument factor**	**Item example/s**	**Instrument factor**	**Item example/s**	**Instrument factor**	**Item example/s**
Withdrawal: psychological	Withdrawal	*I feel stressed if I cannot exercise*	Withdrawal symptoms	*If I have to miss an exercise session, I feel moody and irritable*	Avoidance and rule-driven behavior	*If I cannot exercise, I feel low or depressed*
Mood modification (negative state, general, positive state)	Withdrawal	*I exercise to avoid feeling irritable*	Mood modification	*I use exercise as a way of changing my mood (e.g., to get a buzz, to escape etc.)*	Mood improvement	*I feel happier and/or more positive after I exercise*
Conflict (Interpersonal, other activities)	Reduction in other activities	*My exercise interferes with family responsibilities / My exercise interferes with work/school responsibilities*	Conflict	*Conflicts have arisen between me and my family and/or my partner about the amount of exercise I do*	-	*-*
Salience: cognitive	Reduction in other activities	*I am consumed with thoughts of exercise at home, work, or school*	-	*-*	-	*-*
Salience: general & behavior	Time	*I organize my life around exercise / I spend a great deal of time in exercise related activities*	Salience	*Exercise is the most important thing in my life*	-	*-*
Tolerance	Tolerance	*I continually increase my exercise duration to achieve the desire effects/benefits*	Tolerance	*Over time I have increased the amount of exercise I do in a day*	-	*-*
Continuance despite problems	Continuance	*I exercise despite persistent physical problems*	-	*-*	Avoidance and rule-drive behavior	*I usually continue to exercise despite injury or illness, unless I am very ill or too injured*
Impaired control	Lack of control	*I am unable to reduce how often I exercise*	-	*-*	-	*-*
Impaired control	Intention effects	*I often exercise longer than I intend*	-	*-*	-	*-*
Relapse	-	*-*	Relapse	*If I cut down the amount of exercise I do, and then start again, I always end up exercising as often as I did before*	-	*-*
Catching up on missed exercise	-	*-*	-	*-*	Avoidance and rule-driven behavior	*If I miss an exercise session, I will try and make up for it when I next exercise*
Reason: Body image	-	*-*	-	*-*	Weight and control exercise	*I exercise to burn calories and lose weight*
Withdrawal: Body image	-	*-*	-	*-*	Weight and control exercise	*If I cannot exercise, I worry that I will gain weight*
Exercise as a compensatory behavior	-	*-*	-	*-*	Weight and control exercise	*If I feel I have eaten too much, I will do more exercise*
Lack of enjoyment	-	*-*	-	*-*	Lack of exercise enjoyment	*I do not enjoy exercising*
Rigid exercise pattern	-	*-*	-	*-*	Exercise rigidity	*My weekly pattern of exercise is repetitive*

*EDS, Exercise Dependence Scale; EAI, Exercise Addiction Inventory; CET, Compulsive Exercise Test*.

As shown in Table 2, a total of 33 different components of problematic exercise were identified from the 17 assessment instruments considered in the present study. Fifteen of 33 components were defined based on the six components of addiction (i.e., salience, mood modification, withdrawal, conflict, tolerance, and relapse) proposed by Griffiths (11). Nevertheless, in the present study the salience, mood modification, and withdrawal components were further broken across three domains, while the conflict component was further broken down across four domains.

**Table 2:** Components assessed by psychometric instruments.

**Instrument**	**Body image comparison**	**Catching up on missed exercise**	**Conflict: general**	**Conflict: interpersonal**	**Conflict: intrapersonal**	**Conflict: other activities**	**Continuance despite problems**	**Craving**	**Cross tolerance**	**Exercise as a compensatory behav**.	**Exercise characteristic.: duration**	**Exercise characteristic: frequency**	**Exercise characteristic: type**	**Exercise characteristic: time**	**Exercise reason: affiliation**	**Exercise reason: body image**	**Exercise reason: health**	**Impaired control**	**Lack of enjoyment**	**Mood modification.: unspecified**	**Mood modification: neg. state**	**Mood modification: pos. state**	**Relapse**	**Rigid exercise pattern**	**Salience: behavior**	**Salience: cognitive**	**Salience: general**	**Social norms**	**Striving for control**	**Tolerance**	**Withdrawal: physical**	**Withdrawal: psychological**	**Withdrawal: body image**	***N*°components assessed by the instruments**
CES (22)	°	∙	°	°	°	∙	∙	°	°	°	°	°	°	°	°	°	°	°	°	∙	°	°	°	∙	°	°	°	°	°	°	°	∙	°	6
CPA (23)	°	°	°	°	°	°	°	°	°	°	°	°	°	°	°	°	°	°	∙	°	°	°	°	°	∙	∙	∙	°	°	°	°	°	°	4
CPA-R (24)	°	°	°	°	°	°	°	°	°	°	°	°	°	°	°	°	°	°	∙	°	°	∙	°	°	∙	∙	∙	°	°	°	°	°	°	5
CET (12)	°	∙	°	°	°	°	∙	°	°	∙	°	°	°	°	°	∙	°	°	∙	°	°	∙	°	∙	°	°	°	°	°	°	°	∙	∙	9
EES (25)	°	°	°	°	°	°	∙	∙	°	∙	∙	∙	°	°	°	°	°	°	°	°	°	°	°	∙	°	∙	∙	°	°	°	°	∙	°	9
EAI (10)	°	°	°	∙	°	°	°	°	°	°	°	°	°	°	°	°	°	°	°	∙	°	°	∙	°	°	°	∙	°	°	∙	°	∙	°	6
EAI-R (1)	°	°	°	∙	°	°	°	°	°	°	°	°	°	°	°	°	°	°	°	∙	°	°	∙	°	°	°	∙	°	°	∙	°	∙	°	7
EBQ (26)	°	°	°	°	°	°	°	°	°	°	°	°	°	°	°	°	°	°	°	°	°	°	°	°	°	°	°	°	°	°	∙	∙	∙	3
EDQ (27)	°	°	∙	∙	∙	∙	°	°	°	°	°	°	°	°	∙	∙	∙	∙	°	°	∙	∙	°	∙	°	°	∙	°	°	°	°	∙	°	13
EDS (8)	°	°	°	∙	°	∙	∙	°	°	°	°	°	°	°	°	°	°	∙	°	°	∙	°	°	°	∙	∙	∙	°	°	∙	°	∙	°	10
EDS-R (28)	°	°	°	∙	°	∙	∙	°	°	°	°	°	°	∙	°	°	°	∙	°	°	∙	°	°	°	°	∙	°	°	°	∙	°	°	°	8
ESS (29)	∙	°	°	∙	°	∙	∙	°	°	°	°	∙	°	°	°	∙	°	°	°	°	∙	∙	°	∙	°	∙	°	°	°	∙	∙	∙	∙	14
OEQ (30)	°	∙	°	°	°	∙	∙	∙	∙	∙	°	∙	∙	°	°	°	°	°	∙	°	°	∙	°	°	∙	∙	°	∙	∙	°	°	∙	∙	16
OEQ-1 (31)	°	°	°	°	°	°	°	°	°	∙	°	∙	°	°	°	°	°	∙	°	°	°	°	°	°	°	∙	°	°	°	°	°	∙	∙	6
OEQ-2 (32)	°	°	°	°	°	∙	°	°	°	∙	°	∙	°	°	°	°	°	°	°	°	°	°	°	°	°	∙	°	°	°	°	°	∙	∙	6
OEQ-R (33)	°	°	°	°	°	°	°	°	∙	°	°	∙	∙	°	°	∙	°	°	°	°	°	°	°	°	°	∙	°	°	°	°	°	∙	°	6
PPPE (34)	°	°	∙	∙	∙	∙	∙	°	°	°	°	°	°	∙	°	°	°	∙	°	°	∙	∙	°	°	°	°	°	°	°	∙	°	°	°	10
**Number of instruments assessing the component**	1	3	2	7	2	8	8	2	2	5	1	6	2	2	1	4	1	5	4	3	5	6	2	5	4	10	7	1	1	6	2	13	6	

*CES, Commitment to Exercise Scale; CPA, Commitment Physical Activity; CPA-R, Commitment to Physical Activity Scale Revised; CET, Compulsive Exercise Test; EES, Excessive Exercise Scale; EAI, Exercise Addiction Inventory; EAI-R, Exercise Addiction Inventory Revised; EBQ, Exercise Beliefs Questionnaire; EDQ, Exercise Dependence Questionnaire; EDS, Exercise Dependence Scale; EDS-R, Exercise Dependence Scale Revised; ESS, Exercise Salience Scale; OEQ, Obligatory Exercise Questionnaire; OEQ-R, Obligatory Exercise Questionnaire Revised; PPPES, Problematic Practice of Physical Exercise Scale*.

*∙ Assessed; ° not assessed*.

Other core components of addiction such as impaired control, craving, and cross-tolerance, not explicitly covered by Griffiths' model (11), but referred in other works for behavioral addictions (3, 18, 19, 35), were also identified. Traditional criteria such as the modality or type of exercise, duration of exercise, and frequency of exercise emerged and were grouped together with time to identify the characteristics of exercise that the instruments outlined. In addition, along with time, continuance despite problems was another component identified primarily in the instruments that were based on substance dependence criteria to define problematic exercise. In addition to body-image-related withdrawal, there were five components (i.e., catching up on missed exercise, exercise as a compensatory behavior, body image-related exercise reasons, lack of enjoyment, and rigid exercise pattern) that were mostly identified from instruments which conceptualized exercise as a means to modify weight and body shape. Nevertheless, body image reasons were grouped together with other less frequent components that appeared from items assessing reasons or motives for exercise, such as social relatedness reasons (e.g., “I exercise to meet other people”) or health reasons (e.g., “I exercise to be healthy, feel fit, or prevent heart disease and other illness”). Finally, other components that also had a very low frequency were body image comparison, social norms, and striving for control.

In terms of breadth of coverage, the instruments varied from three to 16 of the 33 identified components (see Table 2). The component most frequently assessed across the instruments in the present review was psychological withdrawal, being more assessed than any other two domains of this component considered in this study: body image-related withdrawal and physical withdrawal. The second most assessed component across the instruments was cognitive salience, which showed a higher presence than general salience, and behavioral salience. The mood modification component, in any of its types, was assessed across 11 instruments. Conflict, in any of its types, was assessed in 10 instruments, although conflict with other activities and interpersonal conflict were assessed more than intrapersonal conflict and general conflict. Among the components common to other behavioral addictions, tolerance, impaired control, overall craving, cross-tolerance, and relapse, were assessed less frequently than any of the aforementioned addiction component groups.

Within the traditional components assessing exercise characteristics, exercise frequency was more assessed in the instruments than exercise time, exercise type, and exercise duration components. However, the continuance despite problems component was more assessed than time within the criteria that were based on substance dependence. There were six components which were presented to a greater or lesser extent in instruments highlighting an obligatory or compulsive character of exercise, being in descending order: body image-related withdrawal, exercise as compensatory behavior, rigid exercise pattern, body image reasons, lack of enjoyment, and catching up on missed exercise. Considered as a whole, 10 of the 17 instruments in the present review assessed one or more of the six aforementioned components. Of these 10 instruments, only the CET (12) assessed all these six components.

## Discussion

Utilizing content analysis, the objective of the present study was to identify, examine, and compare the components of problematic exercise in psychometric instruments assessing problematic exercise identified in a recent systematic review (6). Despite the different theoretical conceptualization, the divergence in the operational definition of their factors, and the variety of ways of wording their items, the instruments reflected some common components that might indicate core criteria when defining and operationalizing problematic exercise.

### Establishing an Operational Definition That Allows the Comparison of Instruments Under the Same and Between Different Theoretical Perspectives

Seventeen self-reported psychometric instruments assessing at least some potential aspect of problematic exercise were reviewed. Prior to the comparison between instruments in terms of their assessed components, a coding and interpretation task was required by the researchers to identify and define the components assessed through the items collected in the instruments. This task did not (in most cases) involve a direct identification of the exercise components, since there are instruments, such as the Commitment to Physical Activity Questionnaire [CPA, (23)] and the Obligatory Exercise Questionnaire (OEQ, 24) that contain a large number of items with diverse content but are encompassed in a one-dimensional structure not defined in the study description (see Appendix B). In other instruments, such as the Commitment to Physical Activity Scale Revised [CPA-R, (24)], the Excessive Exercise Scale [EES, (25)], the Exercise Dependence Questionnaire [EDQ, (27)], and the OEQ-revised [OEQ-R, (33)], the items are grouped into factors, but these are also not defined anywhere in the study description. Finally, in the rest of the instruments, where the items are grouped into factors defined in the study description, inconsistencies were shown between the operational definition of the factors and the wording of the items that assesses the construct in question.

Looking at the comparison between the EDS, EAI, and CET (see Table 1), with a few exceptions (for example, the tolerance component), inconsistencies can be observed between the definition of the factors and the wording of the items intended to assess them. On the one hand, there are factors that in different instruments use the same term, but on further inspection their items assess different components. For example, the EDS and EAI contain a factor assessing withdrawal which, however, show variation in its operational definition (see Appendix B). Thus, some of the items contained in the EDS for withdrawal (i.e., “I feel stressed if I cannot exercise”) reflect the same component defined in the EAI. However, the wording of other items of the EDS included in withdrawal (i.e., “I exercise to avoid feeling irritable”) would reflect the mood modification component defined for the EAI. On the other hand, some factors that are named in the instruments with different terms, actually assess the same component. For example, the time factor in the EDS is defined in a similar way to the salience factor in the EAI. Both factors refer to the dominant role that the exercise plays in the individual's life. These inconsistencies show that instruments that assess problematic exercise utilizing different theoretical conceptualizations, also maintain a lack of consensus when denominating and operationalizing the components of problematic exercise. Therefore, a clear contribution of the present study is to identify the components that assess the items of the instruments, in order to be able to compare the different instruments under the same operational definition of components. In addition, the components and their definitions in the Appendix C represent a code necessary to reproduce or replicate the results of this study.

### Core Components of Problematic Exercise in the Psychometric Assessment Instruments

The components that were most frequently assessed across the items of the instruments reviewed were some of the identified forms of withdrawal (i.e., physical, psychological, and body image), salience (behavioral, cognitive, and general), and mood modification (unspecified, negative state, and positive state). Although no component was assessed by all of the instruments reviewed, this reduced set of components were present in all instrument groups according to the theoretical conceptualization of problematic exercise on which they are based. Despite the use of different terms, there appears to be consensus around these three major components. Therefore, based on the instruments reviewed, it appears that these three components reflect the “core” criteria for problematic exercise. This fact is not surprising because these components are core features of addiction models (10, 11) and have been defined, although sometimes with variations in their terminology, in instruments based on the criteria for substance dependence (8), and in instruments that conceptualize problematic exercise as a means to modify weight and/or body shape (12, 13).

Regarding the withdrawal component, most instruments (*n* = 13) assess the psychological effects of withdrawal, and only two instruments (EES and the Exercise Beliefs Questionnaire [EBQ]) additionally assess the physical effects of exercise cessation. Not surprisingly, psychological withdrawal appears as a core component in problematic exercise instruments, since research has shown that this component is present in other clinically recognized behavioral addictions (i.e., gambling, video gaming) (36–38). However, the low frequency of the physical withdrawal component could be viewed as surprising, especially because (i) some instruments are based on substance dependence criteria, where the physical effects of withdrawal is a defined component, and (ii) literature has previously indicated physical withdrawal effects (e.g., fatigue, heart rate, pain) for potential behavioral addictions, including exercise addiction (2). However, it should be noted that while research has shown the existence of some psychological effects, such as depression or anxiety, resulting from exercise withdrawal (39), the physical effects of withdrawal in the context of exercise have been less studied and is an avenue for future research.

On the other hand, body image-related withdrawal symptoms had a higher frequency in these particular instruments than physical withdrawal, and was assessed primarily in instruments that conceptualized exercise as a means of modifying body shape and weight. Therefore, even though the withdrawal symptoms associated with body image also reflect exercising to avoid negative affect and could reflect this feature as psychological withdrawal, it might well be a common element with other mental health disorders (e.g., eating disorders). Consequently, it would be interesting to incorporate its assessment in instruments that in a comprehensive way evaluate different forms of problematic exercise. In this regard, a differentiation of this type of withdrawal could help to identify different profiles of individuals who present a problematic exercise according to the combination of their symptoms or components (40).

Cognitive salience was the second most assessed component by the problematic exercise instruments (*n* = 10). Some of them, in addition to assessing the cognitive aspect, assess the behavioral aspect of this component, while a few instruments, grouped under a conceptualization of addiction/dependence, assess this component only in a general way (i.e., EAI, EAI-R, and EDQ). In any case, it should be noted that overall salience, referring to strong presence of exercise in the individual's life, was assessed by a number of instruments similar to the withdrawal component (*n* = 13). However, one of the few instruments that does not assess any type of salience is the CET, despite the fact that other instruments grouped under a conceptualization of problematic exercise as a means of modifying the weight and/or body shape assess this component. Future research should examine the role that salience may have in problematic exercise associated with eating and body image disorders.

With respect to the mood modification component, 11 instruments assessed one of the three mood modification forms that emerged, which highlights different definitions of this component. More specifically, instruments that define problematic exercise in terms of behavioral addiction (i.e., EAI and EAI-R) assess this component without going into detail regarding the positive or negative character of the changes in the emotional states experienced as a consequence of exercising. Instruments conceptualizing problematic exercise based on substance dependence criteria (i.e., EDQ, EDS, and EDS-R) assess mood modification in terms of getting relief from a negative emotional state. Finally, instruments conceptualizing problematic exercise as a means of modifying body weight and/or shape (i.e., CET and OEQ) tend to reflect the positive subjective experience in the mood modification component. Based on these results, future research should examine under what circumstances mood modification should be considered a component of problematic exercise. For example, in circumstances where exercise contributes to the relief of a negative state without major significance to individual, it might not be problematic, since the problem would be more what produces the negative subjective experience in individual. However, in those circumstances where exercise behavior is adopted as an almost unique and disproportionate way of dealing with these negative states, this might clearly indicate a problem because this behavior may lead to the exacerbation of other symptoms, such as social isolation or withdrawal. Therefore, future research should determine whether it is valuable to discern between these three components when assessing them in a problematic exercise instrument.

### Candidate Components of Problematic Exercise

Two components, conflict (in some of its forms) and continuance despite problems had a high presence of assessment in the instruments reviewed. However, unlike the three aforementioned core components, these do not appear to be core criteria in the problematic exercise instruments because they are absent in some of the developed instruments according to their conceptualization of problematic exercise.

The conflict component (in its different forms) was assessed in 10 of the 17 instruments reviewed, and was the fourth most frequently assessed component. However, it cannot be considered a “core component” of the instruments because it was absent from assessment instruments that conceptualized problematic exercise as a means of modifying body shape and/or weight (i.e., CET, EES, OEQ and some of the OEQ modifications). In the development of these specific instruments, no form of conflict (i.e., interpersonal, intrapersonal, with other activities) is mentioned as a component of the problematic exercise. Not even in the work that theoretically underpins and develops the CET, the instrument within this conceptualization group that presents a clearer theoretical foundation, can any reference to conflict be found (12, 13).

However, it is surprising that conflict is not assessed in this group of instruments, since recent research suggests the need to consider this component, given that conflict appears to be associated to a greater extent than other components with unhealthy variables associated with eating disorders. For example, Chamberlain and Grant (41) analyzed the symptoms of problematic exercise among individuals with eating disorder traits. Overall, the results of the study showed that the EAI showed a positive association with disordered eating. However, conflict assessed by EAI was the only component associated with emotional dysregulation and obsessive-compulsive personality disorder traits, characteristics that have been attributed to problematic exercise associated with eating disorders (3, 13).

Similarly, Sicilia et al. (42) identified profiles of adolescent exercisers based on exercise addition symptoms assessed with the EAI and examined differences in several health-related variables across these profiles. The results of the Sicilia et al.'s study suggest that conflict may somehow play a key role in differentiating problematic exercise profiles associated with eating disorders (e.g., an eating disorder associated with an emotional state generated by depression or derived from excessive concern for body image). Future research should investigate the role that the conflict component may have in understanding problematic exercise associated with eating disorders.

Continuance despite problems is a relatively frequently assessed component in problematic exercise instruments based on substance dependence criteria (EDQ, EDS, EDS-R) and models that define problematic exercise as a means to modify body shape and/or weight (CET, EES, OEQ). However, this component is not assessed in instruments based on addiction components (e.g., EAI and EAI-R). The continuance despite problems component refers to when an individual continues engaging in exercise despite drawbacks or contraindications to do it, and was highlighted as a consequence of problematic exercise in a case study applying a behavioral addition conceptualization (35). Therefore, along with the conflict component, future studies should analyze the role of continuance despite problems as a possible core symptom of problematic exercise.

### Components Differentiating the Psychometric Assessment Instruments

Except for the three global core components (i.e., withdrawal, salience, and mood modification) and the two candidate components (i.e., conflict, and continuance despite problems), the remaining components had a lesser presence in the assessment instruments reviewed. Tolerance and relapse were two components within the component model for behavioral addictions (11) that had the least presence in the instruments assessing problematic exercise. However, items assessing tolerance were greater than for those assessing relapse, which could be explained by the fact that while tolerance is a component that has been defined both in models of behavioral addictions (11) and substance dependence (3, 8), relapse has only been defined within the first model. In fact, the relapse component was only assessed in the EAI and EAI-R. Both components relate to the body's capacity to adapt to exercise (e.g., need to increase the amount of exercise), so it has been indicated that they may not necessarily reflect a real problem in exercise-specific behavior, especially for elite athletes (1, 43).

Apart from the six core components defined by Griffiths (11) for behavioral addictions, other common addiction components had some inclusion in the instruments (i.e., impaired control, craving, and cross-tolerance). The lower frequency of these components is surprising given that they have all been observed in case study accounts and considered as possible components of behavioral addictions (35), but they have also been considered as criteria for substance dependence in the latest (fifth) edition of the DSM (3). Therefore, it is surprising that impaired control, although assessed in the instruments based on criteria of substance dependence, is not assessed by the EAI and EAI-R, which is limited only to the six core components of behavioral addictions defined by Griffiths (11). However, Griffiths also argued that impaired control was subsumed in the “conflict” component. Even scarcer is the assessment of craving and cross-tolerance which is not assessed in any of the problematic exercise instruments based on either behavioral addiction or substance dependence.

The results show that a relatively small group of instruments assess components that are related to the characteristics of exercise (i.e., types, duration, frequency, time). Among these components, exercise frequency is the most assessed by the instruments, with a greater presence than the duration and time components. The presence of these components is noteworthy, given that literature has repeatedly indicated that the amount of time spent or the form of exercise itself is not a distinctive feature of problematic exercise (1, 44). Therefore, the assessment of these components appears to reflect the initial influence that physical components (i.e., form and mode of exercise) had on the definition of problematic exercise. In fact, the instruments that include the assessment of these components (e.g., time, duration, frequency, etc.) are either instruments based on conceptualizations developed several decades ago (30) or studies that build on the instruments originally proposed in those decades (25, 29) where, along with the assessment of psychological factors, the behavioral components that describe the activity itself are maintained.

However, it is noteworthy that an instrument with a conceptualization of problematic exercise such as the EDS-R includes a time component, restricted to the amount of time the individual spends exercising. This is explained by the fact that the wording of the time component items in the EDS-R do not really capture the operational definition of the construct. More specifically, Hausenblas and Symons-Downs (8) in developing the EDS defined the time factor in line with the criteria defined in the DSM-IV for substance dependence, that is, as “great deal of time is spent in activities necessary to obtain exercise”. In this sense, time is operationalized in the EDS similar to a type of salience (e.g., “I organize my life around exercise”), as defined by the components of behavioral addictions (10, 11). However, the wording of the items in the EDS-R for this factor was changed from the original version (EDS, 8), so that the latter wording, far from capturing the operational definition of the component, reflects more the time that the individual spends on exercise itself (e.g., “I spend a lot of time exercising”).

The DSM considers the criterion of time for substance use disorders, referring to the great deal of time that the individual may spend in obtaining the substance, using the substance, or recovering from its effects (3, 9). Therefore, an adaptation of this criterion, as specified for substance use disorders, to the context of the problematic exercise should be operationalized in relation to the large amount of time per day that the individual spends around exercise (i.e., before, during, and after exercise), and not focus exclusively on the time of exercise performance. A definition in this line is more like a type of behavioral salience than a characteristic of the exercise itself. In fact, exercise time, assessed through frequency or duration, is more concerned with exercise involvement than problematic exercise (44).

In addition to exercise characteristics, reasons or motives for exercise (i.e., social relatedness, body image, and health) are also assessed in some instruments for problematic exercise. The EDQ is the only instrument that assesses these three exercise reasons. As has been indicated for exercise characteristics (i.e., frequency, intensity, type or modality of exercise), research needs to examine whether the motives may themselves reflect characteristics of problematic exercise (44). For example, the motive of exercising for body image reasons was evaluated more frequently than the other two motives, because it was also considered in the instruments that conceptualized problematic exercise as a means of modifying body weight and/or size (i.e., CET, OEQ, OEQ-R). As indicated above, although this group of instruments share components of problematic exercise (i.e., withdrawal, salience, mood modification, continuance despite problems) with other groups of instruments, they nevertheless show clear differences in the assessment of some components. More specifically, catching up on missed exercise, rigid exercise pattern, and lack of enjoyment are components defined in the instruments with a problematic exercise conceptualization as a means to modify body weight and size but has a low frequency of assessment in other instruments with different conceptualization. Moreover, there are clear components (i.e., withdrawal: body image, exercise as a compensatory behavior) that were only present in the instruments that conceptualize problematic exercise associated with body image.

Instruments that conceptualize problematic exercise as a means of modifying body shape and/or weight capture the assessment of components related to concern about body weight and appearance (e.g., withdrawal: body appearance, exercise reason: body image). In addition, these components are absent in the other groups of instruments with different theoretical conceptualizations. Therefore, it is logical to expect that the size of the effect of the relationship found between problematic exercise and eating disorders is larger when it is assessed with instruments that conceptualize problematic exercise as a means to modify the weight and body shape, such as CET, than with instruments under other theoretical conceptualizations (e.g., EAI, EDS), as recent research has found (17, 45). Nevertheless, the assessment instruments for problematic exercise, regardless of their conceptualization of problematic exercise, share assessed components with each other (i.e., withdrawal, salience, mood modification), so it is not surprising to find addictive components present in individuals with eating disorders (16, 41).

### Implications for a Future Consensus on Problematic Exercise Components

The results of the present study reveal a lack of consensus in the operational definition of the components of problematic exercise and a variety of ways of wording their items. This variety of ways of defining problematic exercise makes it difficult to compare results from different assessment instruments. Therefore, a consensus on the components of problematic exercise appears necessary for the advancement of research. The present study contributes, as a first step, in this direction, since the results identify some common components, despite the wide variety of components identified in the instruments. However, although the degree of presence of specific components in the assessment instruments may help to move toward a greater consensus on the operational components of problematic exercise, this should not be the only criterion to be considered. There are several issues that should be taken into account in the future.

First, there is a need for specific criteria, based on empirical and/or clinical research (e.g., medical case studies), to support the components to be evaluated through the items in psychometric assessment instruments. The development of some of the instruments reviewed in the present study show no clear theoretical conceptualization, while other instruments have proposed components of problematic exercise considering features in other behavioral addictions and substance use disorders, but also in other disorders that could be associated with problematic exercise (6). However, it should be noted that the screening of problematic exercise through psychometric assessment instruments is limited without the definition of diagnostic criteria.

Second, those components that showed lower frequencies in the assessment instruments reviewed in the present study should not be classified *a priori* as peripheral components of problematic exercise. It should be noted that some of them may well reflect the variety of conceptualizations used in the instruments. On the other hand, it must be assumed that problematic exercise is a complex phenomenon, because it may involve various forms of expression and can occur in individuals who exercise in different ways and for different reasons. This diversity could be approached from different theoretical perspectives. Therefore, an approach that highlights the differences will be directed to the development of instruments that assess a specific manifestation of problematic exercise. An approach that highlights the similarities between the different manifestations of problematic exercise will focus on assessing only the core components of this phenomenon [see for example the model of common components to behavioral addictions proposed by Griffiths, (11, 46)]. Far from somewhat antagonistic proposals, a third possibility would be to propose comprehensive conceptualizations that contemplate the development of instruments that include both core components of the various manifestations of problematic exercise and some of its differentiated components. Along these lines, Sicilia et al. (6), based on the proposal of Shaffer et al. (40), suggested a broader conceptualization that considers problematic exercise as a broad family of different expressions that are individually distinguished by the specific contribution of their factors. Although none of these three approaches should be considered as better than the others, nevertheless, each of them illuminates the development of problematic exercise instruments and the components that should be included.

Third, there is a wide consensus that a behavior becomes problematic when it is harmful or has negative consequences for individual (1, 6, 8, 47). Therefore, taking into account the aforementioned considerations, a key issue in selecting the components that should define problematic exercise is that they should reflect the pathological nature of the behavior, and therefore include components that are necessarily negative (46, 48). A practice that includes a large number of components without sufficient evidence would fall into the risk of overpathologizing exercise behavior. Components that do not express a functional impairment, psychological distress, or a clear separation from normative behavior in context should not be components to be included in instruments of assessment for problematic exercise (49). For example, the time component, referring to the amount of time an individual spends exercising, has been indicated as a characteristic that in the specific exercise behavior probably does not reflect a problem in itself, and produces confusion when differentiating problematic exercise from high exercise involvement (44).

Finally, in the development of instruments, authors should take special care in the wording of the items in order to capture, as precisely as possible, the operational definition of the problematic exercise component they are trying to assess. Therefore, test developers should prevent the opposite practice described in the previous paragraph whereby components, reflecting some potential damage of the exercise, nevertheless in the wording of the items that assess this component do not capture this quality. As Griffiths (46) pointed out, some components that he adopted from Brown (50) for his model of behavioral addictions clearly reflect the negative aspect. However, this aspect may not have been reflected in some of the items used in the assessment instruments for behavioral addictions. For example, as Griffiths points out, the original concept of salience offered by Brown refers to “when the particular activity becomes the most important activity in the person's life and dominates their thinking (preoccupations and cognitive distortions), feeling (cravings) and behavior (deterioration of socialized behavior)...even if the person is not actually engaged in the behavior they will be thinking about the next time they will be” [(46), p. 180]. In this sense, the original concept clearly focuses on the negative aspects of behavior, through experiencing cognitive distortions, and a total cognitive preoccupation, along with a deterioration of the individual's socialization.

However, the content analysis of the items in the instruments that assess this component for problematic exercise, as suggested by Griffiths, does not always reflect a negative element of the behavior for the individual.

Focusing on the instruments analyzed in the present review, we found wording of items such as “I look forward to physical activity” (e.g., CPA, CPA-R), “How often do you think about exercise?” (e.g., EES), “Exercise is the most important thing in my life” (e.g., EAI, EAI-R), “I organize my life around exercise” (e.g., EDS), “Exercise is frequently on my mind” (e.g., ESS), and “I have had daydreams about exercising” (e.g., OEQ, OEQ-1, OEQ-2, OEQ-R). Although all of these items may reflect the salience component, they clearly are not reflecting the negative character that Griffiths (11, 46) refers to.

Therefore, a re-evaluation is needed when reviewing the instruments in order to reach consensus on the inclusion of components that should define the problematic exercise in all its different manifestations. On the one hand, based on further empirical and clinical evidence, components that do not reflect the problematic nature of the behavior should be excluded from future instruments by assessing this construct. On the other hand, the items should be written in such a way that they clearly reflect the negative component of this construct, therefore avoiding either the instrument overpathologizing individuals who exercise, or clearly harmful components being omitted by inappropriate wording of the items assessing the components.

## Limitations

This review addresses for the first time a compilation and comparison of the components present in the psychometric instruments currently available that assess problematic exercise. Nonetheless, several limitations of the present study should be highlighted. First, following the approach adopted in the systematic review previously conducted by the present authors (6), instruments assessing problematic exercise in specific exercise or sport contexts (e.g., dance, running, bodybuilders) or adaptations of existing instruments in a new language or culture were not included. Consequently, the possibility exists that some other components specifically proposed for these contexts may not have been captured in the present study. Second, the components emerged from studies that, in some cases, were developed among samples that might have included some proportion of non-exercising individuals (e.g., university students, secondary school students). Finally, the review of instruments was limited to studies written in languages spoken by the authors of the present study (i.e., English and Spanish).

## Conclusions

Despite the disparity of operational definitions and instruments proposed for the assessment of problematic exercise, components such as withdrawal, salience, and mood modification appear to be present in all the groups of instruments considered. Consequently, these might well form the “core” group of components of problematic exercise. Despite being present in many of the instruments, components such as conflict and continuance despite problems are clearly absent in one of the groups of instruments. That is, conflict is absent in the group of instruments than concern body image, while continuance despite problems is absent in those that are based on addiction criteria. Finally, a wider number of components of differing nature appears to be specific to the variety of conceptualizations used in the currently available instruments. In view of the disparity of potential components of problematic exercise identified in the present study, and in the interest of reaching a consensus that allows to advance in this research field, further studies are needed to resolve which of those components could be considered to be inherently problematic.

## Author Contributions

ÁS designed the study, conducted the content analysis, and performed initial drafts of the manuscript. MA-I conducted the content analysis and contributed to the drafting of the manuscript and revisions. AP contributed to the drafting of the manuscript and revisions. MG designed the study, conducted the content analysis, and contributed to the drafting of the manuscript and revisions. All authors assisted with drafting of the final version of the manuscript, including critical revisions for intellectual content, contributed to the article, and approved the submitted version.

## Funding

This research is part of the I+D+I Project (Grant Number PID2019-107674RB-I00), funded by Ministerio de Ciencia e Innovación (MCIN), Agencia Estatal de Investigación (AEI/10.13039/501100011033), Spain. MA-I (UAL RRA202101) is funded by Ministero de Universidades (Plan de Recuperación, Transformación y Resiliencia, Next Generation EU). AP (FPU18/01055) is funded by MCIN/AEI/10.13039/501100011033 and Fondo Social Europeo (FSE). ‘El FSE invierte en tu futuro'.

## Conflict of Interest

The authors declare that the research was conducted in the absence of any commercial or financial relationships that could be construed as a potential conflict of interest.

## Publisher's Note

All claims expressed in this article are solely those of the authors and do not necessarily represent those of their affiliated organizations, or those of the publisher, the editors and the reviewers. Any product that may be evaluated in this article, or claim that may be made by its manufacturer, is not guaranteed or endorsed by the publisher.
