# Supplementary material for: A Review of the Components of Problematic Exercise in Psychometric Assessment Instruments
Source: Front Public Health. 2022 Mar 31;10:839902. doi: 10.3389/fpubh.2022.839902 (PMC9008204; doi:10.3389/fpubh.2022.839902)
Supplement: Supplementary file 1 [file Table_1.DOCX]

**Supplementary material:**

**A Review of the Components of Problematic Exercise in Psychometric Assessment Instruments**

- **Appendix A.** Systematic Review Coding Sheet
- **Appendix B.** Characteristics, structure, and factors in the psychometric instruments assessing problematic exercise
- **Appendix C**. Components, definition and example of item categorized through the evaluation of the psychometric assessment instruments

Appendix A. Review Coding Sheet

**Instrument**

Insert name measure

**Authors**

Insert citation and year of study

**Sample size**

Mean age

Mean BMI

Gender

Characteristics

**Conceptualization**

1. Problematic exercise as end of a continuum of exercise
2. Problematic exercise as a behaviour to regulate body shape and weight
3. Problematic exercise as a behavioural addiction
4. Problematic exercise as a dependence
5. No clear conceptualization

**Instrument Structure**

Number items and factors

**Components and Definition**

Insert name factor

Insert definition factor by authors

Appendix B. Characteristics, structure, and factors in the psychometric instruments assessing problematic exercise

| Instrument | Authors | Sample size (characteristics) | Conceptualization | Instrument Structure | Factors and Definition |
| --- | --- | --- | --- | --- | --- |
| Commitment to exercise scale (CES) | Davis et al. (1993) | 185 Exercisers recruited from recreational facilities at university, health and fitness clubs and associations in Canada Men (*N*=88; mean age=28.93; SD=9.42)  Women (*N*=97; mean age=26.71; SD=8.81) | Problematic exercise as end of a continuum of exercise | 8 items with 2 factors | 1) Obligatory: aspect of exercising whereby psychological well-being is contingent upon assiduous adherence to a regular and structured exercise regimen  2) Pathological: when exercise is continued in the face of adverse circumstances, and when it tends to take precedence over the social component of one's life |
| Commitment to Physical Activity questionnaire (CPA) | Corbin et al. (1987) | 450 College students enrolled in PE classes at an USA University (Men=238; Women=212) | Problematic exercise as end of a continuum of exercise | 12 items with unidimensional structure | ------- |
| Commitment to Physical Activity Scale –Revised (CPA-R) | DeBate et al. (2009) | 937 Girls, aged 8 to 13, from different locations across USA taking part in an PA intervention program | Problematic exercise as end of a continuum of exercise | 12 items with 3 factors | 1) Value of PA (Nd)  2) Attitudes toward PA (Nd)  3) Motivation regarding PA (Nd) |
| Compulsive Exercise Test (CET) | Taranis et al. (2011) | 367 young women (Mage=20.76, SD=2.39, range=18-30), recruited from a UK university (68,8%) and Australian university (28,1%) engaged in regular exercise or sport over the last 4 weeks (M=4.27 h/w).  BMI=21.86 (SD=2.77; range=16.3-38.2) | Problematic exercise as a behaviour to regulate body shape and weight | 24 items with 5 factors: Avoidance and rule-driven behaviour; | 1) Avoidance and rule-driven behaviour: reflects rule-driven behaviour (e.g., making up for missed sessions, continued exercise despite injury, experiencing guilt and feelings of having let the self down when unable to exercise) and continued exercise to avoid affective withdrawal symptoms.  2) Weight control exercise: related to exercising for weight and shape reasons and the use of dysfunctional weight control practices.  3) Mood improvement: reflects the positive reinforcement component of exercise in terms of mood.  4) Lack of exercise enjoyment: reflects experiencing exercise as a chore and deriving no enjoyment from it.  5) Exercise rigidity: reflects a rigid behavioural pattern in terms of exercise |
| Excessive Exercise Scale (EES) | McCabe & Vincent (2002) | 413 secondary schools’ students (*Boys*=221; Mage=13.76, SD=1.07; Girls=192; Mage=13.81, SD=1.10) | Problematic exercise as a behaviour to regulate body shape and weight | 8 items with 2 factors: | 1) Need for exercise (Nd)  2) Focus on exercise (Nd) |
| Exercise Addiction Inventory (EAI) | Terry et al. (2004) | 200 university students,  (102 sport science students; 98 psychology students), age from 18-40, who reported regular participation in exercise.  (Mage=21.24, SD=3.77); Men=111 (Mage=20.82); Women=189 (Mage=21.75) | Problematic exercise as a behavioural addiction | 6 items with unidimensional structure. Each item reflects a component | 1) Salience: when the particular activity becomes the most important activity in the person’s life and dominates their thinking (preoccupations and cognitive distortions), feelings (cravings), and behaviour.  2) Mood modification: subjective experiences that people report as a consequence of engaging in the particular activity and can be seen as a coping strategy.  3) Tolerance: process whereby increasing amounts of the particular activity are required to achieve the former effects.  4) Withdrawal symptoms: unpleasant feeling states and/or physical effects which occur when the particular activity is discontinued or suddenly reduced, e.g., the shakes, moodiness, irritability etc.  5) Conflict: refers to the conflicts between the addict and those around them (interpersonal conflict), conflicts with other activities (job, social life, hobbies and interests) or from within the individual themselves [intrapsychic conflict] which are concerned with the particular activity.  6) Relapse: tendency for repeated reversions to earlier patterns of the particular activity to recur and for even the most extreme patterns typical of the height of the addiction to be quickly restored after many years of abstinence or control. |
| Exercise Addiction Inventory (EAI-R) | Szabo et al (2019) | 277 young and adult individuals (Men=243; Women=34; aged from 22 to 45) recruited on social media and exercised regularly at least three times per week | Problematic exercise as a behavioural addiction | 6 items with unidimensional structure. Each item reflects a component | 1) Salience (Ada);  2) Mood modification (Ada);  3) Tolerance (Ada);  4) Withdrawal symptoms (Ada);  5) Conflict (Ada);  6) Relapse (Ada). |
| Exercise Beliefs Questionnaire (EBQ) | Loumidis & Wells (1998) | 13 exercisers (Male=7; Female=6; aged from 21 to 40) recruited from a university sports centre and who reported exercised over three times a week. | Problematic exercise as a dependence | 21 items with 4 factors | 1) Social desirability: people’s concerns about becoming socially inadequate, inferior or undesirable if unable to exercise.  2) Physical appearance: maladaptive beliefs about exercise concerned with physical appearance or the fear of becoming physically unattractive if unable to exercise.  3) Mental and emotional functioning: concerns about the effect that exercise cessation could have on mental and emotional functioning.  4) Vulnerability to disease and ageing: concerns about  being more vulnerable to disease, growing old early, or one’s brain becoming unhealthy. |
| Exercise Dependence Questionnaire (EDQ) | Ogden et al. (1997) | 449 young and adult participants (Male=161; Mage=32.85; Female=288; Mage=31.26) recruited from sports clubs, leisure centres, and ads in magazines, reported exercising more than 4 hours/week. | Problematic exercise as a dependence | 29 items with 8 factors | 1) Interference with social / family / work life: Nd  2) Positive reward: Nd  3) Withdrawal symptoms: Nd  4) Exercise for weight control: Nd  5) Insight into problem: Nd  6) Exercise for social reasons: Nd  7) Exercise for health reason: Nd  8) Stereotyped behaviour: Nd |
| Exercise Dependence Scale (EDS) | Hausenblas & Symons-Downs (2002) | 266 university students (57,7% men; Mage=21.72, SD=2.89 | Problematic exercise as a dependence | 31 items with 7 factors | 1) Tolerance: need for increased amounts of exercise to achieve the desired effect or diminished effect with continued use of the same amount of exercise.  2) Withdrawal: manifested by either the characteristic withdrawal symptoms for exercise or the same [or closely related] amount of exercise is engaged in to relieve or avoid withdrawal symptoms.  3) Intention effects: exercise is often taken in larger amounts or over a longer period than was intended.  4) Lack of control: there is a persistent desire or unsuccessful effort to cut down or control exercise.  5) Time: a great deal of time is spent in activities necessary to obtain exercise.  6) Reduction in other activities: social, occupational, or recreational activities are given up or reduced because of exercise.  7) Continuance: exercise is continued despite knowledge of having a persistent or recurrent physical or psychological problem that is likely to have been caused or exacerbated by the exercise |
| Exercise Dependence Scale-Revised (EDS-R) | Symons-Downs et al. (2004) | 408 university students (65.7% women; Mage = 20.2 years, SD = 2.5) participating in fitness classes at least three times per week | Problematic exercise as a dependence | 21 items with 7 factors: | 1) Tolerance: Ada  2) Withdrawal: Ada  3) Intention Effects: Ada  4) Lack of Control: Ada  5) Time: Ada  6) Reduction in Other Activities: Ada  7) Continuance: Ada |
| Exercise Salience Scale (ESS) (a) | Kline, Franken, & Rowland, (1994) | 74 university students (Men=32, Women=42) enrolled in undergraduate psychology courses (Mage=23.17; SD=6.31). | No clear conceptualization | 40 items with 2 major factors and 4 minor factors undefined | 1) Response Omission Anxiety: experiences a dysphoric or anxious mood when unable to exercise.  2) Response Persistence: persists in exercise behaviour in the face of physical consequences, such as bad weather and physical injury. |
| Obligatory Exercise Questionnaire (OEQ) | Pasman & Thompson (1988) | 90 volunteers, aged 18-60, 15 men and 15 women in each of the three following groups: obligatory runners (Mage women=33.1, Mage men=37.2); obligatory weightlifters (Mage women=27.4, Mage men=26.7); sedentary group (Mage women=29.1; Mage men=32.3). | Problematic exercise as a behaviour to regulate body shape and weight | 20 items with unidimensional structure | ------ |
| Obligatory Exercise Questionnaire (OEQ-1) | Steffen & Brehm (1999) | 250 high school students (Women=133; Men=117) | Problematic exercise as a behaviour to regulate body shape and weight | 10 items with 3 factors: | 1) Emotional element of exercise: items concerning negative emotional consequences of failing to exercise.  2) Exercise frequency and intensity: items concerning one’s personal sense of being compelled or driven to exercise  3) Exercise preoccupation: items describing someone who thinks a lot about exercising. |
| Obligatory Exercise Questionnaire (OEQ-2) | Ackard et al, (2002) | 586 female university students (Mage=20.61; SD=3.09). Actual BMI=22.79; SD=4.51. Ideal BMI=20.31; SD=2.17. | Problematic exercise as a behaviour to regulate body shape and weight | 11 items with 3 factors | 1) Exercise fixation: items describing a preoccupation  with exercise, negative affect associated with missed exercise, and the use of exercise to compensate for perceived overeating.  2) Exercise frequency: items describing the frequency and type of exercise episodes.  3) Exercise commitment: items indicative of an individual’s sense that routine exercise episodes cannot be missed. |
| Obligatory Exercise Questionnaire – Revised (OEQ-R) | Duncan et al. (2012) | 241 exercisers (Men=143 Mage=29.95 SD=11.12; Women=97, Mage=32.89, SD=12.47; 1 case did not report gender). | Problematic exercise as a behaviour to regulate body shape and weight | 10 items with 3 factors | 1) Preoccupation with exercise: Nd  2) Exercise behaviour: Nd  3) Exercise emotionally: Nd |
| Problematic Practice of Physical Exercise Scale (PPPE) | Kotbagi et al. (2015) | 341 leisure exercisers (Men=232; Women=109) involved in activities such as yoga, cricket, soccer, gymnastics, swimming, tennis and dancing (Mage=28.26; SD=10.83) | No clear conceptualization | 25 items (from EDQ and EDS-R) with 6 factors and 4 subfactors | 1) Lack of control: reflect the individual’s incapacity to decide his exercising habits.  2) Stereotypical behaviour: 2a) intention (determination to act in a certain  Way); and 2b) continuity (the fact that the same behaviour is continuous in time, recurring frequently or at times even without interruption).  3) Motivation for health: 3a) physical health (Nd), and 3b) psychological health (Nd).  4) Withdrawal: uncomfortable physical or mental changes that happen when the body is deprived of a substance that it is accustomed to getting. Here, the changes are attributed to the deprivation of exercise.  5) Interference with social life: similar to reduction of other activities in EDS-R.  6) Tolerance: reflects the individual’s capacity to challenge himself and endure more hardships while exercising. |

*Note:* PE = Physical Education; USA = United States of America; UK = United Kingdom; BMI = Body Mass Index; PA = Physical Activity; DSM = Diagnostic and Statistical Manual of Mental Disorders; Nd = not defined by the authors; Ada = As defined above

Appendix C. Components, definition and example of item of the components measured by the psychometric instruments

| **Component** | **Definition** | **Example of item** |
| --- | --- | --- |
| Body image comparison | Refers to when individual confronts their body image to other people | I am aware of my size and shape and a gaze at my refection in mirrors and windows more frequently than most people do |
| Catching up on missed exercise | Refers to the need to exercise after missing a session or workout | If I miss a planned workout, I attempt to make up for it the next day |
| Conflict: General | Refers to global negative consequences of exercise that affects the individual’s life in a general way, without specifying any particular life’s domain | My exercising is ruining my life |
| Conflict: Interpersonal | Tension/conflict between the individual and those around them (e.g., friends, family, and partner) that occurs, or could occur, as consequence of exercise | I would end a relationship if it prevented me from exercising |
| Conflict: Intrapersonal | Tension/conflict within individual themselves as consequences of their exercise | I feel guilty about the amount I exercise |
| Conflict: Other activities | Refers to when individual report actual or possible interferences between exercise and other life’s activities (e.g., work/school responsibilities, social invitations) | My level of exercising makes me tired at work |
| Continuance despite problems | Refers to when individual continues doing exercise despite drawbacks or contraindications to do it | I often exercise despite injury, fatigue or mild illness |
| Craving | Refers to the subjective experience of intense desire to engage in exercise | Sometimes, I feel a need to exercise twice in one day, even though I may feel a little tired |
| Cross tolerance | Refers to the need to do other sport modalities if individual cannot do the habitual one | I will engage in other forms of exercise if I am unable to engage in my usual form of exercise |
| Exercise as compensatory behaviour | Refers to when individual exercise, or intends to, as a mean to compensate other behaviours (e.g., overeating) | If I feel I have eaten too much, I will do more exercise |
| Exercise characteristic: Duration | Refers to the time spent exercising within a given period of time | On average, how long do you exercise on each occasion? |
| Exercise characteristic: Frequency | Refers to the number of times the individual exercises within a given period of time | I exercise at least four days every week |
| Exercise characteristic: Type | Refers to the type (or types) of exercise that is performed | I engage in one/more of the following forms of exercise: walking, jogging/running or weightlifting |
| Exercise characteristic: Time | Refers to great deal of time that individual spends doing exercise | I spend a lot of time exercising |
| Exercise reason: Social relatedness | Refers to when individual exercises to make friends or avoid being alone | I exercise to meet other people |
| Exercise reason: Body image | Refers to when individual exercises to control or modify the body appearance, specially shape and weight | I exercise to look attractive |
| Exercise reason: Health | Refers to when individual exercises to keep, improves their health or prevents any diseases | I exercise to be healthy |
| Impaired control | Lack or decreased individual’s control over exercise reflected by engaging in the behaviour even when they do not want to, or engaging in more frequency, intensity or longer duration than intended | I am unable to reduce how often I exercise |
| Lack of enjoyment | Refers to when individual experience exercise as a chore and they do not feel before and/or during the activity as an enjoyable experience. | I do not enjoy exercising |
| Mood modification: Unspecified | Refers to feeling o mood changes experienced by individual during and/or after exercising without any specification of the either positive or negative character of these experiences | I use exercise as a way of changing my mood (e.g. to get a buzz, to escape etc.) |
| Mood modification: Negative state | Relief from an unpleasant or negative subjective state is obtained as a consequence of exercising | After an exercise session I feel less anxious |
| Mood modification: Positive state | Pleasant or positive subjective experience that individual has as a consequence of exercising | I have experienced a feeling of euphoria or a “high” during or after an exercise session |
| Relapse | Refers to the tendency to repeat the same (or greater) amount of exercise after a certain time without doing the activity or after withdrawal from the activity (e.g., due to injury). | If I cut down the amount of exercise I do, and then start again, I always end up exercising as often as I did before |
| Rigid exercise pattern | Refers to when individual follow a rigid pattern to exercise (e.g. follow a plan regularly) | I follow a set routine for my exercise sessions e.g. walk or run the same route, particular exercises, same amount of time, and so on |
| Salience: Behaviour | When exercise becoming the most important thing in the individual’s life so that the own behaviour aimed at ensuring a next exercise session | I would arrange or change my schedule participate in physical activity |
| Salience: Cognitive | Refers to strong presence of exercise in the individual’s mind | Sometimes, I find that my mind wanders to thoughts about exercising |
| Salience: General | Refers more generally to the importance of exercise in the individual’s life without no references to a particular thinking or behaviour | Physical activity is the high point in my day |
| Social norms | Reflects the social influence from other significant (e.g., on what they think or do about exercise) which could affect the individual’s exercise behaviour. | My best friend likes to exercise |
| Striving for control | Individual try to keep a control about their load of training in order to improve their performance | I keep a record of my exercise performance, such as how long I work out, how far or fast I run / I frequently “push myself to the limits” |
| Tolerance | Refers to the need to increase the amount of exercise to achieve the desired effects or benefits | I continually increase my exercise frequency to achieve the desire effects/benefits |
| Withdrawal: Physical | Unpleasant physical effects that individual manifests when exercise is suddenly reduced or stopped (e.g., Insomnia, sluggish) | If I miss a day of exercise, I feel as if my muscles have atrophied |
| Withdrawal: Psychological | Unpleasant feeling states reported by individual when exercise is reduced or stopped (e.g., irritated, anxious, stressed) | If I cannot exercise I feel irritable |
| Withdrawal: Body image | Unpleasant feeling states by a negative perception of the body's appearance and reported by individual when exercise is reduced or stopped (e.g., outside the socially sanctioned body ideal) | When I miss an exercise session, I feel concerned about my body possibly getting out of shape |
